# The Spectrum of Human Herpesvirus 8/Epstein–Barr Virus-Co-Positive Lymphoproliferations and Lymphomas

**DOI:** 10.3390/diseases14090323

**Published:** 2026-09-05

**Authors:** Epameinondas Koumpis, Maria Nasiou, Georgios Monastiriotis, Dimitrios Leonardos, Vasileios Georgoulis, Elisavet Apostolidou, Alexandra Papoudou-Bai, Panagiotis Kanavaros, Eleftheria Hatzimichael

**Affiliations:** 1 Department of Hematology, Faculty of Medicine, School of Health Sciences, University of Ioannina, 45500 Ioannina, Greece; ekoumpis@outlook.com (E.K.); marianasiou13@gmail.com (M.N.); geomon913@gmail.com (G.M.); d.leonardos@uoi.gr (D.L.); v.georgoulis@uoi.gr (V.G.); e.apostolidou@uoi.gr (E.A.); 2 German Medical Institute, 1, Nikis Avenue, 4108 Limassol, Cyprus; alexandra.papoudou@goc.com.cy; 3 Department of Anatomy-Histology-Embryology, Faculty of Medicine, School of Health Sciences, University of Ioannina, 45110 Ioannina, Greece; pkanavar@uoi.gr

**Keywords:** human herpesvirus 8, Kaposi sarcoma–associated herpesvirus, Epstein–Barr virus, co-infection, primary effusion lymphoma, germinotropic lymphoproliferative disorder, multicentric Castleman disease, B-cell lymphoma

## Abstract

Background/Objectives: Human herpesvirus 8 (HHV-8), also known as Kaposi sarcoma-associated herpesvirus (KSHV), is a gamma-2 herpesvirus implicated in a distinctive group of lymphoproliferative disorders (LPDs) and lymphomas. In some of these entities, lesional cells are concurrently infected with Epstein–Barr virus (EBV), a gamma-1 herpesvirus, raising important questions regarding viral cooperation in lymphomagenesis. This narrative review summarizes the clinicopathological spectrum and biological significance of HHV-8/EBV co-positive lymphoproliferations. Methods: We provide a narrative synthesis of the biology of HHV-8 and EBV, including latent, abortive lytic, and productive lytic infection programmes and viral mechanisms involved in cell-cycle deregulation, apoptosis inhibition, immune evasion, and B-cell transformation. Major HHV-8-associated lymphoproliferative entities are reviewed within the current WHO-HAEM5 and International Consensus Classification frameworks, with particular attention to patterns of EBV co-infection and diagnostically atypical or overlapping lesions. Results: Primary effusion lymphoma, including its extracavitary presentation, is frequently EBV-positive, whereas HHV-8-positive germinotropic lymphoproliferative disorder is characteristically HHV-8/EBV dual-positive. In contrast, HHV-8-positive lesional cells in multicentric Castleman disease and HHV-8-positive diffuse large B-cell lymphoma are typically EBV-negative. True dual positivity requires demonstration of HHV-8 latency-associated nuclear antigen and EBV-encoded RNA within the same morphologically defined lesional cell population. Rare atypical cases show overlapping clinicopathological features among established entities. Conclusions: HHV-8/EBV co-positive lymphoproliferations comprise a biologically and diagnostically heterogeneous group. Although unusual overlapping cases suggest potential relationships among HHV-8-associated disorders, current evidence does not support a single continuous disease spectrum or a uniform mechanism of HHV-8/EBV cooperation.

## 1. Introduction

The human herpesvirus-8 (HHV-8) was identified as the causative agent of Kaposi sarcoma (KS) in 1994 [1]. HHV-8, also designated Kaposi sarcoma-associated herpesvirus (KSHV/HHV-8), has since been implicated in several distinct lymphoproliferative disorders (LPD) and lymphomas with characteristic clinical and histopathological features, including a propensity for co-infection with Epstein–Barr virus (EBV) [2,3,4]. Specifically, KSHV/HHV-8 has been identified in multicentric Castleman disease (MCD) [5], in the rare germinotropic LPD (GLPD) [6], in primary effusion lymphoma (PEL) and its solid variant called extracavitary PEL (EC-PEL) [7,8], and in HHV-8-positive diffuse large B-cell lymphoma, not otherwise specified [9]. In PELs and GLPD the neoplastic lymphoid cells additionally harbor EBV, a virus pathogenetically linked to 1–2% of all human cancers worldwide [6,7]. PEL and KSHV/HHV8-positive GLPD are the principal recognized settings in which EBV may be demonstrated within KSHV/HHV8-positive lesional cells. In contrast, KSHV/HHV8-associated MCD and KSHV/HHV8-positive DLBCL are usually EBV-negative, although rare dual-positive or diagnostically overlapping lesions have been reported [10,11,12]. These cases raise important questions regarding classification, cellular viral distribution, and possible biological relationships among KSHV/HHV8-associated lymphoproliferative disorders. The present review examines these lesions within the WHO-HAEM5 and ICC frameworks, distinguishing confirmed or strongly supported lesional dual positivity from concurrent viral detection in separate or incompletely characterized cell populations.

With the exception of GLPD, most of these disorders arise in human immunodeficiency virus (HIV)-positive patients [12,13] although cases in HIV-seronegative individuals have also been reported [14,15]. Notably, occasional cases with atypical and overlapping features across these entities have been described, pointing to a grey zone within the HHV-8/EBV co-positive LPD spectrum that warrants further study and characterization [10,11]. The present review summarizes the spectrum of HHV-8/EBV co-positive lymphoproliferations.

## 2. Biology of HHV-8

### 2.1. HHV-8

HHV-8, classified as a gamma-2 herpesvirus, is primarily known for its association with KS. It is also called Kaposi sarcoma-associated herpesvirus (KSHV) [16]. It shares phylogenetic and functional similarities with EBV, a member of the gamma-1 herpesvirus subfamily. A defining characteristic of gammaherpesviruses is their association with neoplastic processes, as evidenced in both animal models and experimental systems [17]. More precisely, KSHV/HHV-8, a lymphotropic virus, is linked to multiple forms of lymphoproliferative disorders and lymphomas [9]. These include PEL and its solid variant extracavitary PEL, MCD, DLBCL, and the rare entity called GLPD [9,16]. In addition, Kaposi sarcoma inflammatory cytokine syndrome (KICS) is a recently characterized clinical entity observed in HIV-positive individuals co-infected with KSHV/HHV-8 [18,19]. It is distinguished by markedly elevated levels of interleukin-6 (IL-6) and high HHV-8 viral loads in affected patients [18,19]. Saliva, blood, and sexual contact are the three main ways the virus spreads, while the global seroprevalence of KSHV/HHV-8 is estimated to range from 5% to 20% [20]. The highest seroprevalence rates, surpassing 50%, are observed in Africa and the Brazilian Amazon [21]. Despite its widespread occurrence, a limited number of individuals who are infected advance to the manifestation of diseases associated with KSHV/HHV-8, except those demonstrating impaired immune functionality [20,21].

### 2.2. HHV-8 Life Cycle

HHV-8 is identified as a double-stranded DNA virus, with a genomic length that varies from 165–170 kb [22,23]. Like other herpesviruses, HHV-8 has two distinct phases in its life cycle: latent and lytic replication [20,22,24]. Following the initial infection, the virus remains in a dormant state as an episome and depends on the host’s cellular mechanisms for its replication process. More precisely, B lymphocytes are the main viral target, while endothelial and epithelial cells represent an alternative site for viral latency; endothelial ones constitute the principal infected cell type in KS [20].

Latency is characterized by a low gene expression rate, while the virus utilizes the host cellular DNA polymerase for its genome maintenance and distribution to daughter cells; there is no virion production in this phase [20,22,24]. The contribution of latent proteins to oncogenesis is of particular importance, as they help infected cells survive and proliferate [22]. The principal latent genes transcribed during KSHV/HHV-8 infection include latency-associated nuclear antigen (LANA), viral cyclin (v-Cyclin), viral FLICE-inhibitory protein (vFLIP), and K12/Kaposin (encompassing Kaposin A, B, and C), along with approximately 25 mature microRNAs (miRNAs) [17]. In addition, within HHV-8-associated MCD and PEL tissues, an additional latent gene, viral interferon regulatory factor 3 (vIRF3), has been identified [17,22]. vIRF3 plays a critical role in suppressing interferon signaling pathways, thereby promoting cellular proliferation and enhancing cell survival [17,22].

LANA, the most widely expressed latent protein in both latently infected cells and HHV-8-associated tumors, is about 1162 amino acids in length [16]. LANA represents a pivotal multifunctional protein in the preservation of HHV-8 latency and oncogenesis, as it impacts several cellular signaling pathways and proteins, including replication factors, transcription factors, and chromatin-modifying enzymes [20,25]. The control and deregulation of crucial signaling pathways such as MAPK, JAK/STAT, ERK, PI3K/AKT, Notch, and Wnt by LANA enable a successful latent infection [20,25]. Furthermore, LANA enhances cell cycle progression through the inactivation of the p53 and Rb (retinoblastoma) tumor suppressor proteins, thereby impairing the apoptotic mechanisms in HHV-8-infected cells [20,22,25]. V-Cyclin is responsible for the regulation of the cell cycle and cell proliferation by activating cellular cyclin-dependent kinase 6 (CDK6) [26]. The vCyclin-CDK6 complex plays a critical role in the phosphorylation and subsequent inactivation of the Rb protein, thereby facilitating the process of tumorigenesis [20,26,27].

Another key protein essential for the survival and proliferation of HHV-8-infected cells is viral FLICE-inhibitory protein (vFLIP) [28]. Its primary function involves the constitutive activation of the NF-κB signaling pathway, which in turn upregulates the transcription of anti-apoptotic genes, including members of the BCL-2 family [20,29]. Kaposins constitute a group of latent proteins, which includes kaposin A, B, and C [30,31]. Within this group, Kaposin B facilitates tumorigenesis through the upregulation of cytokine expression [20]. Cytokines act as pivotal mediators in oncogenic mechanisms, and a variety of inflammatory cytokines are released in response to HHV-8 infection, thereby establishing a microenvironment that favors tumor progression [20]. IL-6 and IL-10, in particular, are identified as autocrine growth factors for MCD and PEL [20]. HHV-8 miRNAs have been shown to target and inhibit the expression of numerous cellular and viral genes, thereby controlling virus life cycles, cell immune response, virus-induced angiogenesis, and the spread of HHV-8 [16]. Approximately 25 mature miRNAs are expressed in KS or MCD infected cells and are detected in different levels in separate phases of virus life cycle [32,33,34,35]. More details about the functions of miRNAs are beyond the scope of this text.

Although latency and productive lytic replication represent useful conceptual endpoints, KSHV/HHV-8 gene expression is not invariably binary. During abortive lytic replication, a restricted subset of immediate-early and/or early lytic genes may be expressed without completion of the full lytic cascade or production of infectious virions. This intermediate gene-expression state may occur in a minority of cells within otherwise predominantly latent KSHV/HHV-8-associated tumours. The resulting lytic gene products may nevertheless contribute to tumorigenesis through both cell-intrinsic and paracrine mechanisms, including modulation of DNA-damage responses, cell survival, cytokine and chemokine signalling, angiogenesis, and immune evasion [36].

By contrast, productive lytic replication involves coordinated activation of the complete lytic gene cascade, viral DNA replication, expression of late structural genes, and the assembly and release of infectious virions, thereby enabling viral replication and dissemination [16,24]. The transition from latency to lytic replication is regulated by multiple factors, including inflammation, hypoxia, oxidative stress, and epigenetic modifications. However, the immune status of the host appears to be the most critical determinant of reactivation. Immunosuppression, particularly in the context of co-infections with viruses such as HIV, HSV, HHV-6, and CMV, has been shown to precipitate the shift from latent to lytic replication [16,20,37]. The lytic genes associated with HHV-8 contribute to oncogenesis by modifying DNA repair pathways, promoting cellular longevity, and facilitating immune system evasion. Some of the most important ones are v-IL6, v-BCL2, v-MIP, v-GPCR, and viral IFN regulatory factor (v-IRF-1) [37]. Most of their functions have been extensively investigated and are now well characterized. For instance, v-IL6 serves as a crucial factor in the development and expansion of B-cells [37]. In addition, vIRF-1 disrupts the DNA damage response by inhibiting the activation of ATM, an upstream protein kinase that activates p53 and promotes cell-cycle arrest in response to DNA damage, and v-GPCR facilitates angiogenesis by stimulating various signaling pathways that are inactivated during latency, including MAPK, PI3K/AKT, and NF-κB, resulting in the upregulation of angiogenic mediators such as vascular endothelial growth factor (VEGF), IL-6, and platelet-derived growth factor (PDGF) [29,37]. Furthermore, both v-GPCR and v-IRF-1 enhance apoptosis resistance by stimulating the NF-κB signaling pathway and suppressing pro-apoptotic factors, respectively [29].

## 3. Biology of EBV

The Epstein–Barr Virus (EBV), alternatively referred to as human herpesvirus 4 (HHV4), is also classified as a member of the gammaherpesvirus family [38]. The initial detection of EBV was documented in 1964 in Burkitt lymphoma, and subsequent investigations revealed its association with various additional lymphomas, including classical Hodgkin lymphoma and non-Hodgkin lymphoma in post-transplant recipients and individuals affected with HIV, as well as T-cell lymphoma and NK/T cell lymphoma [20,38,39,40,41,42,43]. EBV has also been implicated in several types of cancer in epithelial cells such as nasopharyngeal carcinoma and gastric carcinoma [38,44]. In addition to its correlation with various human malignant neoplasms, EBV is also associated with non-malignant conditions such as infectious mononucleosis, oral hairy leukoplakia, systemic lupus erythematosus, and multiple sclerosis [20,38].The prevalence of EBV infections is usually obtained during the initial years of life through salivary transmission and approximately more than 90% of global population have been exposed to EBV once in their life [20]. EBV is classified as a linear, double-stranded DNA virus with a genome length of about 171 kb which is circularized in latently infected cells [44,45]. These cells are memory B lymphocytes, and it is well established that EBV infection typically remains asymptomatic in individuals with a competent immune system [38]. However, various contributing factors have been implicated in the development of EBV-associated malignancies, including genetic mutations, immune deficiencies, immunosuppression, and co-infection with HIV [38]. Like other herpesviruses, EBV presents two separate phases in its life-cycle; latency and lytic replication [38,44,46]. During latency, the virus remains within the nucleus as a circular episome, which is intricately connected to the chromatin of the host genome via the activity of a viral protein known as EBNA-1 [38]. EBNA-1 is the only EBV-encoded protein consistently expressed across all latency programmes and is therefore detected in virtually all EBV-associated malignancies [47]. In the context of latency, the virus exhibits expression of a limited subset of viral genes alongside non-coding RNAs and relies upon the standard cellular division mechanisms, which encompass the host’s DNA replication apparatus to facilitate the passive replication of its viral genome, thereby ensuring transmission to daughter cells [38]. Latency is a defining characteristic of EBV, as the virus establishes a lifelong latency within human hosts, characterized by infrequent episodes of reactivation and lytic replication [38]. Depending on the microenvironment the virus encounters, EBV can adjust its gene expression program, resulting in different types of latent EBV infection. The most crucial determining factors of this adjustment are cell-target phenotype and the host’s immune response [44,47,48]. More details about these latency types are beyond the scope of this review.

To enter B cells, EBV must interact with their CD21 receptor via its surface protein known as gp350 and, at the same time, with HLA class II molecules via gp42 protein [20,38]. During primary infection, EBV enters a latent phase by expressing only a few specific genes such as nuclear antigens EBNA-1, 2, 3A, 3B, 3C and leader protein (LP), latent membrane proteins LMP-1, 2A, 2B, EBV-encoded small RNAs EBER-1 and 2 and some miRNAs [20,44,47].

EBNA-1, as the only protein expressed in all EBV-associated malignancies, is crucial for the replication and preservation of the EBV genome and may function as an oncogenic factor [47,49]. EBNA-1 plays a critical role in promoting cell survival, even in the presence of DNA damage, by inhibiting the p53-dependent activation of p21 and the associated apoptotic signaling pathways [20,47]. It further enhances the anti-apoptotic function by downregulating the expression of the myc oncogene while simultaneously promoting the expression of anti-apoptotic proteins such as BCL2 and survivin [50]. EBNA-1 has been implicated in the upregulation of NOX2, the catalytic subunit of NADPH oxidase, which plays a central role in the production of reactive oxygen species (ROS). This increase in ROS contributes to genomic instability by inducing chromosomal aberrations, DNA damage, and telomere dysfunction [20,51].

EBNA-2 acts as a transcriptional activator of many viral and cellular genes, such as LMP-1, LMP-2A, MYC, CD21, and CD23, which are essential for the proliferation and immortalization of B lymphocytes. More precisely, EBNA-2 synergistically interacts with EBNA-LP and can stimulate the expression of cyclin D2 within resting B lymphocytes; cyclin D2 represents a vital constituent of the cellular cycle machinery and promotes the progression of the cell cycle during the initial G1 phase, thus preventing their apoptosis [20,47]. EBNAs-3 (A, B, and C) proteins present an anti-apoptotic functionality spectrum, including the inhibition of the accumulation of CDK (cyclin-dependent kinase) inhibitors, the degradation of the tumor suppressor protein Rb, the stabilization of the c-myc oncogene, and the suppression of pro-apoptotic proteins [20,38].

LMP-1, a transmembrane protein with CD40 receptor properties, activates cell signaling pathways such as NF-κB, MAPK/ERK, PI3K/AKT, Notch, and JAK/STAT and therefore leads to oncogenesis by several mechanisms [20,38,47,52]. The most critical hallmarks include the induction of genomic instability, resistance to apoptosis, unlimited replicative capacity, the reprogramming of energy metabolism, promotion of tumor-associated inflammation, the facilitation of tissue invasion and metastasis, and the evasion of DNA damage checkpoint mechanisms [20]. Another anti-apoptotic protein, LMP-2, mainly promotes cellular transition into the S phase. It upregulates the survivin protein expression, enhances the cyclin-E expression, and activates a specific tyrosine kinase signaling that helps the tumor to survive [20,47].

EBV-encoded small RNAs (EBER-1 and EBER-2) are identified as non-translated RNAs and are acknowledged as trustworthy indicators for the in situ hybridization method applied to detect EBV infection in clinical specimens of various tumors such as gastric carcinoma, lymphoma, and nasopharyngeal carcinoma, among others [47]. Their well-documented function is the enhancement of the G1 to S cycle phase transition by activating the oncogenic PI3K/Akt signaling pathway and inhibiting the tumor suppressor PKR and the cell cycle inhibitors p21 and p27 and, as a result, protecting EBV-infected cells from apoptosis [20,38,47].

Under certain conditions, latently infected EBV-positive cells may undergo viral reactivation. Potential triggers include hypoxia, oxidative stress, temperature fluctuations, epigenetic alterations, and stimulation by various biological agents [43]. Productive lytic replication is initiated principally by the immediate-early transcription factors BZLF1, also known as Z, Zta, or ZEBRA, and BRLF1, also known as R or Rta. These factors activate early lytic genes required for viral DNA replication, which is followed by late structural gene expression and the assembly and release of infectious virions [38,44,46,48].

However, EBV reactivation does not invariably progress to productive replication. Abortive lytic replication denotes the expression of a restricted or non-canonical subset of lytic genes without completion of the full lytic cascade or production of infectious virions. For example, BZLF1 expression has been demonstrated in EBV-positive peripheral T-cell lymphomas displaying a predominantly latency II transcriptional profile, consistent with sporadic abortive lytic activation. More recently, EBV-positive central nervous system lymphoproliferative diseases were shown to express the EBV lytic kinases BXLF1 and BGLF4, but not BZLF1, supporting an incomplete lytic programme associated with locus-specific epigenetic activation. These observations demonstrate that selected lytic proteins may be expressed in otherwise predominantly latent EBV-associated neoplasms without infectious virion production [36,53,54].

Abortively expressed lytic proteins may contribute to oncogenesis through cell-autonomous mechanisms, including interference with DNA-damage responses, apoptosis, proliferation, and antigen presentation, as well as through non-cell-autonomous effects mediated by cytokines, chemokines, angiogenic factors, and remodelling of the tumour microenvironment. Thus, detection of a lytic gene product should not automatically be interpreted as evidence of complete viral replication. This distinction may also have therapeutic relevance, because expression of viral kinases such as BXLF1 and BGLF4 may provide a mechanistic basis for susceptibility to antiviral-based treatment even in the absence of a fully productive lytic cycle.

There is an intricate relationship between the immune system and the viruses in a continuous struggle for dominance. The first one is pivotal in managing EBV infection by generating specific antibodies targeting EBV proteins with the purpose of eliminating it from the body, and the second one exhibits the ability to evade the immune response through various mechanisms, thereby enabling it to establish a chronic infection within the host [38,47,48]. Additionally, EBV can disrupt the functionality of the immune system in multiple ways, thereby complicating the ability of the body to combat the infection: downregulation of MHC class I and II genes, obstruction of the transporter of antigen processing, inhibition of the processing and presentation of cytotoxic T-cell epitopes, and competition with type I interferon signaling are among the ways that further research must be carried out in order to further understand the oncogenesis in EBV-infected cells [46,48].

## 4. KSHV/HHV8-Associated Lymphoproliferative Disorders and Dual-Positive Lesions

HHV-8 is implicated in a range of LPDs, including PEL and its solid variant extracavitary PEL, MCD, DLBCL-not otherwise specified, and the rare GLPD. Importantly, neoplastic cells in both PEL and GLPD are frequently co-infected with EBV. Furthermore, occasional cases displaying atypical or overlapping clinical and histopathological features among these disorders have been documented, suggesting that the complete spectrum of HHV-8-associated LPD is not yet fully delineated.

### 4.1. Current Classification and Operational Definition of Dual Positivity

The fifth edition of the World Health Organization Classification of Haematolymphoid Tumours (WHO-HAEM5) and the International Consensus Classification (ICC) recognize several distinct KSHV/HHV8-associated lymphoid proliferations and lymphomas, including primary effusion lymphoma (PEL), KSHV/HHV8-positive germinotropic lymphoproliferative disorder (GLPD), KSHV/HHV8-associated multicentric Castleman disease (MCD), and KSHV/HHV8-positive diffuse large B-cell lymphoma (DLBCL). Although these conditions share KSHV/HHV8 infection and may show overlapping plasmablastic morphology, they remain distinct clinicopathological entities or processes and should not be interpreted as a single disease solely on the basis of viral positivity [9,55].

WHO-HAEM5 classifies KSHV/HHV8-associated MCD among tumour-like lesions with B-cell predominance and emphasizes that its diagnosis requires the integration of clinical, histological, haematological, immunological, and virological findings. By contrast, PEL and KSHV/HHV8-positive DLBCL are classified as aggressive lymphomas, while GLPD is a usually indolent lymphoproliferative disorder with a characteristic germinotropic distribution. The relationship with EBV also differs among these entities. EBV is detected in most, but not all, cases of PEL and is characteristic of most reported cases of GLPD. Conversely, the KSHV/HHV8-positive plasmablasts of MCD and KSHV/HHV8-positive DLBCL are usually EBV-negative. Rare lesions with features of MCD, GLPD, PEL, or large B-cell lymphoma and concurrent EBV positivity have nevertheless been reported [10,11,12,56,57,58,59,60,61,62].

For the purposes of this review, dual positivity refers to the demonstration of KSHV/HHV8 and EBV within the same morphologically defined lesional cell population. KSHV/HHV8 infection is usually demonstrated by immunohistochemical expression of latency-associated nuclear antigen 1 (LANA-1), whereas EBV is most reliably detected by in situ hybridization for EBV-encoded small RNAs (EBER). Same-cell dual positivity is most securely established by double-labelling or multiplex methods. When these are unavailable, convincing morphological and topographical correspondence between LANA-positive and EBER-positive atypical cells on serial sections may support lesional dual positivity, although the level of evidence should be stated.

The presence of LANA-positive and EBER-positive cells in the same tissue does not necessarily establish coinfection of the same cells. Accordingly, lesions in which the two viruses are demonstrated in separate cell populations—or in which their cellular distribution cannot be determined—are described here as showing concurrent tissue positivity rather than confirmed same-cell coinfection. Similarly, EBV serology, circulating EBV DNA, KSHV/HHV8 viral load, or detection of either virus outside the lesional tissue does not establish dual positivity of the lymphoproliferative lesion.

**Figure 1:**
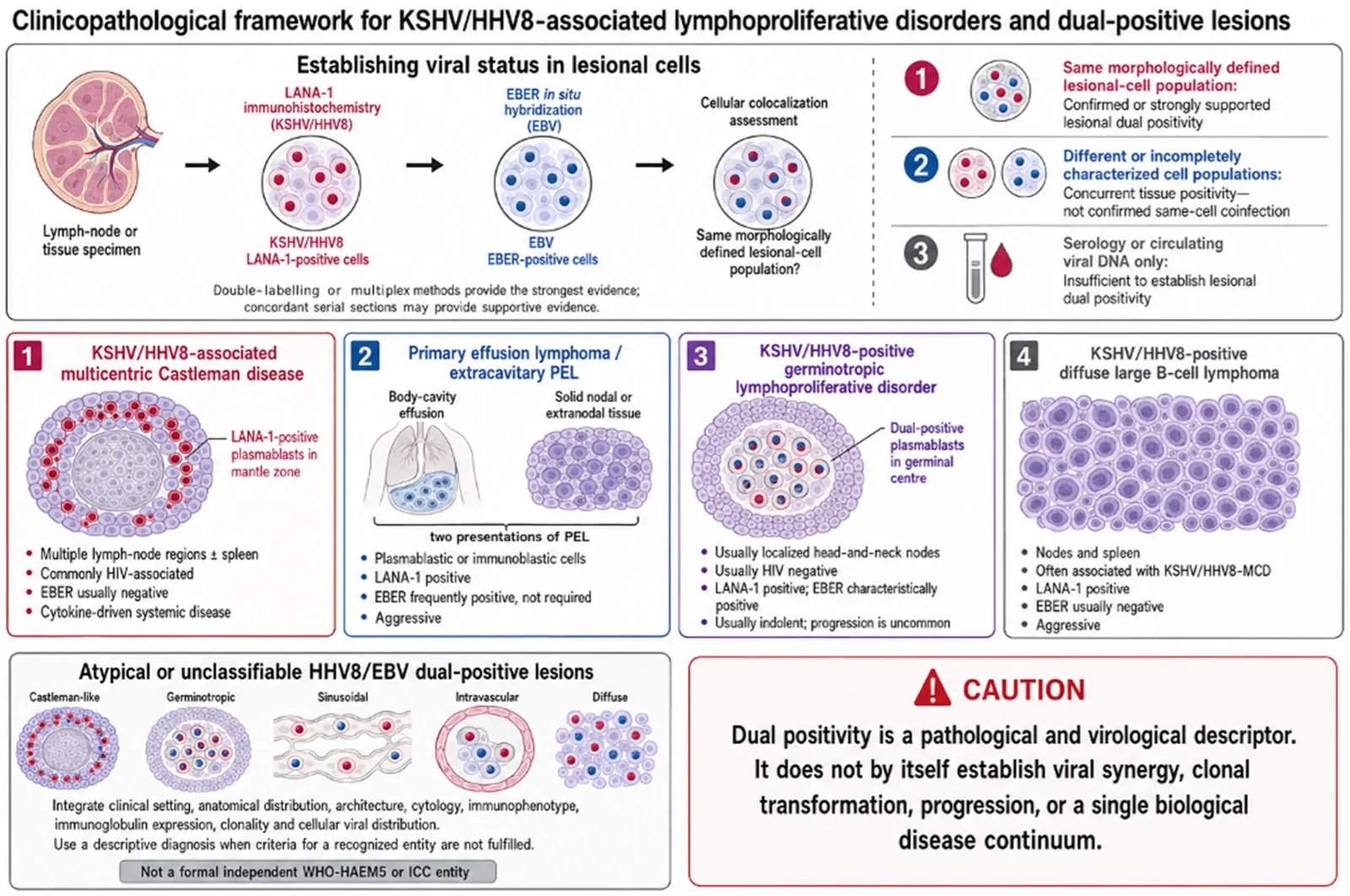
Clinicopathological framework for KSHV/HHV8-associated lymphoproliferative disorders and dual-positive lesions. The recognized entities differ in anatomical distribution, architecture, clinical setting and typical EBV status. Lesional dual positivity requires demonstration of LANA-1 and EBER within the same morphologically defined cell population. Concurrent detection of the viruses in different or indeterminate cell populations does not establish same-cell coinfection. Atypical dual-positive lesions require integrated assessment and may be reported descriptively when the criteria for a recognized WHO-HAEM5 or ICC entity are not fulfilled. Dual positivity does not itself demonstrate viral cooperation or a biological disease continuum.

Atypical dual-positive lesions should therefore be classified through an integrated assessment of clinical presentation, immune-dysregulation setting, anatomical distribution, tissue architecture, cytological features, immunophenotype, immunoglobulin expression, clonality, and cellular viral distribution. When the diagnostic criteria for a recognized WHO-HAEM5 or ICC entity are not fulfilled, a descriptive diagnosis that records the morphology, both viral associations, and the clinical setting may be more appropriate than forced assignment to a specific category. Importantly, dual positivity is a pathological and virological descriptor and does not, by itself, establish a distinct disease entity, clonal transformation, or functional cooperation between the two viruses. WHO-HAEM5 recognizes KSHV/HHV8-associated MCD, KSHV/HHV8-positive GLPD, PEL and its extracavitary presentation, and KSHV/HHV8-positive DLBCL as distinct clinicopathological processes, while acknowledging that occasional cases demonstrate overlapping features. The operational assessment of viral colocalization and its integration with the current WHO-HAEM5 and ICC classification framework are illustrated in Figure 1, while the principal clinicopathological characteristics and typical EBV status of these disorders are summarized in Table 1.

All KSHV/HHV8-associated lesions require demonstration of viral infection in the relevant lesional cells, generally by LANA-1 immunohistochemistry. In this review, “dual positivity” denotes detection of LANA-1 and EBER within the same morphologically defined lesional-cell population. Detection of the two viruses in different or indeterminate cell populations, or detection based only on serology or circulating viral DNA, does not establish lesional-cell coinfection. The designation “emerging KSHV/HHV8–EBV dual-positive large-cell lymphoma” is used provisionally to facilitate collective consideration of recurring cases and does not represent an independent WHO-HAEM5 or ICC disease entity.

### 4.2. Potential Cooperative Pathogenesis of KSHV/HHV8 and EBV

KSHV/HHV8 and EBV encode proteins and non-coding RNAs that influence several overlapping cellular pathways, providing biological plausibility for cooperative effects in dual-infected B cells. However, same-cell coinfection does not by itself demonstrate functional synergy, and the contribution of EBV may differ substantially among PEL, GLPD, and emerging dual-positive large-cell lymphomas [63].

Experimental models provide evidence that KSHV/HHV8 and EBV can cooperate functionally rather than merely coexist within the same cells. In humanized mice, dual infection enhanced KSHV persistence in EBV-transformed B cells; dual-infected cells displayed a plasma-cell-like gene-expression profile resembling PEL, accompanied by increased EBV lytic gene expression and greater tumour formation than with EBV infection alone [64]. A subsequent humanized-mouse study showed that KSHV/EBV dual infection also reshaped innate immunity by promoting the accumulation of a poorly cytotoxic CD56-negative NK-cell population with impaired activity against susceptible and antibody-opsonized targets [65]. These findings identify complementary mechanisms of cooperation: EBV supports expansion of the B-cell reservoir in which KSHV can persist, KSHV enhances EBV lytic gene expression, and dual infection weakens immune surveillance. Thus, experimental evidence supports a cancer-driving role for viral cooperation in at least some settings, particularly PEL-like lymphomagenesis. Nevertheless, same-cell dual positivity in a human lesion does not by itself establish functional synergy, and the extent to which these mechanisms apply to GLPD and emerging dual-positive large-cell lymphomas remains uncertain.

At the molecular level, the viral proteins and non-coding RNAs encoded by both viruses promote cell-cycle progression, inhibit apoptosis, reprogramme host gene expression, and suppress immune responses. EBV expresses latency proteins such as LMP1, which activates the NF-κB and JAK/STAT pathways to promote B-cell proliferation and survival, and LMP2A, which mimics B-cell receptor signalling. EBV also encodes EBERs and BHRF1 miRNAs that inhibit apoptosis and induces widespread epigenetic reprogramming through hypomethylation of host DNA, leading to increased gene-expression variability—a hallmark of cancer [63]. Moreover, EBNA1 evades recognition by cytotoxic T lymphocytes by limiting its own translation and proteasomal processing, thereby reducing antigen presentation [66].

KSHV similarly maintains latency using proteins like LANA, which tethers viral DNA to host chromosomes and inhibits tumor suppressors like p53 and Rb, while also suppressing protein degradation and translation, helping it to remain under the immune radar; vFLIP, which activates NF-κB to prevent apoptosis; and vCYC, a viral cyclin that drives cell cycle progression [63,66]. An additional KSHV protein, vIL-6, likely contributes to inflammatory and hyperplastic changes in lymphoid tissues, while the localized expression of vIL-6 and other inducible viral proteins such as vIRF1 suggests that KSHV manipulates cytokine signaling to shape a microenvironment favorable for disease progression [67]. Other KSHV proteins such as Kaposin B also support tumorigenesis in specific settings [63]. During predominant latency, KSHV restricts global viral protein expression to reduce immune recognition. Nevertheless, selected inducible or lytic gene products, including vIL-6, vIRF1, and MIR1/MIR2, may be expressed through partial or abortive lytic programmes without completion of productive viral replication. Such proteins may promote cell survival, impair antigen presentation and interferon signalling, and condition a cytokine-rich and immunosuppressive microenvironment. Accordingly, expression of a lytic protein in a KSHV/HHV8-associated lesion should not be equated with infectious virion production [36,67]. Abortive lytic replication therefore provides a mechanism through which selected lytic products of either virus could contribute to the biology of otherwise predominantly latent dual-positive lesions.

KSHV also produces a cluster of microRNAs from the Kaposin locus that target cellular genes involved in cell cycle arrest, apoptosis, and immune regulation. Notably, KSHV miR-K12-11 mimics cellular miR-155, an oncogenic miRNA, and both viruses incorporate miRNAs into virions, enabling immediate impact upon infection [63].

In co-infected B cells or in the context of HIV/AIDS, the oncogenic effects of EBV and KSHV may be amplified. Both viruses converge on NF-κB signaling, redundantly block apoptosis, and drive B-cell proliferation through viral protein expression and miRNA-mediated mimicry of host pathways. HIV contributes indirectly to this process by inducing severe immunosuppression, allowing latent viral gene expression to go unchecked, and by fostering a pro-inflammatory environment that supports chronic B-cell activation. While EBV is found in about 30% and KSHV in 5% of AIDS-related lymphomas (ARLs), most cases are virus-negative, raising questions about additional unknown oncogenic mechanisms driven by HIV itself. Nonetheless, the shared and complementary strategies used by EBV and KSHV in infected B cells create a highly favorable environment for malignant transformation, particularly under conditions of immune dysfunction. This understanding has already informed therapeutic strategies that target virus-specific proteins or exploit the oncogenic pathways these viruses manipulate [63].

### 4.3. Primary Effusion Lymphoma (PEL) and Extracavitary PEL

Primary effusion lymphoma (PEL) is an aggressive and uncommon form of B-cell non-Hodgkin lymphoma linked to KSHV/HHV-8 [56]. It predominantly arises in body cavities such as the pleural, peritoneal, and pericardial spaces but can also involve the subarachnoid space and blood vessels [56]. The extracavitary (EC) variant of PEL presents as solid tumors in lymph nodes, the gastrointestinal tract, and the skin while maintaining the genetic and immunophenotypic characteristics of classic PEL [57]. The locations where PEL can develop vary, with cases reported in the subarachnoid space, suggesting that PEL can arise in areas without mesothelial linings [57]. Remarkably, one case involved a woman who developed PEL in an artificial cavity near a silicone breast implant, demonstrating that PEL can emerge in spaces that are not naturally occurring anatomical structures [57]. Additionally, variations in the spectrum of changes associated with multicentric Castleman disease have also been observed [57].

PEL/EC-PEL primarily affects HIV/AIDS patients, accounting for 2% to 4% of AIDS-related lymphomas [56]. It is also observed in immunosuppressed individuals, such as post-transplant patients, and in rare HIV-negative cases, particularly among elderly individuals [57]. HIV-positive individuals with PEL/EC-PEL are often co-infected with EBV, with cases typically occurring in men with a median age of 42 years and CD4 counts below 200 cells/μL [57]. In contrast, HIV-negative PEL cases more frequently express occasional B-cell markers and are less likely to harbor EBV, often presenting in older adults (70–80 years) with poorer outcomes [56]. Notably, advanced age itself may contribute to immunodeficiency [57].

Regarding the clinical presentation, symptoms of PEL arise due to malignant effusions or an EC mass, leading to dyspnea, abdominal distention, and chest pain, depending on the location of the tumor. KSHV also induces immune dysregulation through elevated levels of huIL-10, huIL-6, and vIL-6, contributing to systemic symptoms such as fever, cachexia, edema, and anemia [56]. Many patients present with concurrent or prior Kaposi sarcoma (KS) or KSHV-associated multicentric Castleman disease (KSHV-MCD), with 33% of PEL cases associated with KSHV-MCD and up to 75% having KS [56]. Severe immune dysregulation can lead to Kaposi sarcoma inflammatory cytokine syndrome (KICS), significantly worsening prognosis [56]. EC-PEL can develop before or after classic PEL, particularly in HIV-positive patients [10]. Regarding the pathological findings of PEL, PEL/EC-PEL lymphoma cells are large, pleomorphic, and exhibit plasmablastic or immunoblastic features, occasionally resembling Reed–Sternberg or anaplastic large-cell lymphoma cells [56]. The defining feature of PEL/EC-PEL is HHV-8 positivity, confirmed by expression of latency-associated nuclear antigen (LANA). Despite their B-cell origin, these tumors typically lack B-cell markers such as CD19, CD20, CD22, CD79a, and PAX5, with rare immunoglobulin expression. Some cases show lambda or, less commonly, kappa light chain positivity. They also lack germinal center markers (CD10, BCL6) but express plasma cell differentiation markers (IRF4/MUM1, BLIMP1/PRDM1, CD38, CD138) and activation markers like CD30 (70–80% of cases). Aberrant T-cell antigen expression (e.g., CD3, CD4) has been reported, with rare cases showing monoclonal T-cell populations [56]. Genetic analysis reveals somatic hypermutations in immunoglobulin genes and mutations in proto-oncogenes (BCL6, MYC, PAX5, RhoH/TTF), while TP53 mutations are typically absent. The APOBEC mutational signature, indicative of viral influence, is frequently observed [56]. Gene expression profiling places PEL/EC-PEL within the plasmablastic lymphoma spectrum, suggesting its derivation from terminally differentiated B cells [56]. EBV, when present, follows a latency I gene expression pattern [56]. Cytologic examination of effusion fluid is the primary diagnostic method, with tumor cells exhibiting a plasmablastic or anaplastic morphology and strong LANA-1 expression. Routine LANA-1 immunostaining is essential for distinguishing PEL/EC-PEL from other lymphomas, particularly in HIV-positive or immunosuppressed patients [58]. Differential diagnoses include plasmablastic lymphoma, ALK+ large B-cell lymphoma, and HHV-8-positive DLBCL. Additionally, GLPD, a localized HHV-8-associated lymphoproliferation with a germinal center distribution, must be considered in differential diagnoses due to its potential for progression to aggressive lymphoma [58]. Notably, PEL usually lacks cytoplasmic immunoglobulin expression [68].

Treatment of PEL is primarily based on multi-agent chemotherapy, often with antiretroviral therapy (ART) in HIV-positive patients. The most common regimens include dose-adjusted EPOCH (etoposide, prednisolone, vincristine, cyclophosphamide, doxorubicin) or CHOP (cyclophosphamide, doxorubicin, vincristine, prednisolone). Rituximab may be used if CD20 is expressed, though this is rare [69]. ART is crucial, with studies showing nearly double overall survival when combined with chemotherapy. In a single institution study of 51 patients, ART-based regimens yielded a high complete remission rate and a median survival of 10 months, compared to 3–6 months in early studies without ART. CHOP-like regimens—with or without high-dose methotrexate—have shown complete remission rates of 40–60%, although methotrexate demonstrated no survival benefit [70]. Some remissions have been achieved with ART alone [56]. The prognosis of PEL/EC-PEL remains poor, with <50% of patients surviving beyond one year [69]. Poor prognostic factors include ECOG performance status > 2, low CD4 counts, elevated lactate dehydrogenase (LDH), and multiple body cavity involvement [56]. Interestingly, HIV status does not significantly impact prognosis. Molecular-targeted therapies, including immunomodulatory drugs (thalidomide, lenalidomide, pomalidomide), proteasome inhibitors (bortezomib), and monoclonal antibodies (brentuximab for CD30, daratumumab for CD38), have been explored but with limited success [69].

### 4.4. Kaposi Sarcoma-Associated Herpesvirus/Human Herpesvirus 8-Positive Germinotropic Lymphoproliferative Disorder (KSHV/HHV-8-Positive GLPD)

Kaposi sarcoma-associated herpesvirus/human herpesvirus 8-positive germinotropic lymphoproliferative disorder (KSHV/HHV-8-positive GLPD) is a rare disorder first described in 2002 by Du et al., marked by plasmablasts co-infected with KS-associated HHV-8 and EBV, primarily affecting GCs of B-cell follicles [71]. The disease predominantly affects males in their 40s, 50s, and 60s [72]. Except for one case, all reported GLPD cases involved neoplastic cells co-infected with HHV-8 and EBV, though the precise role and mechanisms of these viruses in disease pathogenesis remain unclear. Limited genetic analyses have not identified established pathogenic driver mutations; this observation is compatible with, but does not prove, predominantly virus-mediated pathogenesis [73]. Clinically, patients typically present with localized, slow-growing lymphadenopathy, most commonly in the head and neck region, often persisting for 3 to 10 years before diagnosis [59,72]. Symptoms such as fever, fatigue, weight loss, effusion, leg swelling, abdominal pain, and paresthesia were observed in some cases [72]. Splenomegaly was present in 4 cases, while all evaluated bone marrow biopsies were negative [72]. Excisional lymph-node biopsy is generally preferred because assessment of architecture is important [72]. Most cases occur in HIV-negative individuals, but in HIV-positive cases, the disease may present with generalized lymphadenopathy, B symptoms, and progression to high-grade lymphoma [74]. Among the four HIV-positive cases reported, two achieved complete remission, one had persistent disease, and another experienced disease progression and died [72].

Additional conditions observed among patients with KSHV/HHV-8-positive GLPD included hepatitis C virus (HCV) infection, with one patient also presenting with hepatitis B virus (HBV) co-infection and autoimmune hemolytic anemia. Comorbidities such as angina pectoris, chronic obstructive pulmonary disease (COPD), and liver cirrhosis were also noted. Progression to high-grade lymphoma was also noted [73].

Histopathological analysis reveals that affected lymph nodes maintain either a preserved or partially disrupted architecture with loosely nodular infiltration of large abnormal cells resembling plasmablasts. These cells preferentially localize within and replace germinal centers. Plasmablasts may also extend into mantle zones, interfollicular regions, or sinuses. Follicular hyperplasia and interfollicular polytypic plasmacytosis are common accompanying findings [73]. Atrophic and hyalinized follicles resembling Castleman disease have been observed in some cases [68].

KSHV/HHV-8-positive GLPD is distinguished from MCD by the co-infection of HHV-8 and EBV. A study by Nabel et al. detected EBV transcripts and EBER-positive cells in HHV8-positive MCD lymph node samples; however, cellular colocalization with LANA-positive plasmablasts was not established. These findings, therefore, indicate concurrent tissue positivity rather than confirmed same-cell dual positivity [75]. Unlike MCD, KSHV-associated GLPD is characterized by plasmablasts co-infected with EBV, capable of expressing any heavy or light chain, preferentially targeting germinal centers, and containing mutated immunoglobulin genes [71].

Immunohistochemically, plasmablasts are positive for HHV-8 and EBV, as determined by in situ hybridization for EBV-encoded RNA (EBER), although EBV-negative GLPD has also been reported [60]. The typical immunophenotype consists of negativity for some B-cell markers (CD20, CD79α, PAX5) and CD138, with variable expression of MUM1/IRF4, CD38, and CD30. Aberrant CD3 expression was observed in 6 cases despite the absence of other T-cell markers [72]. A subset of cases showed weak expression for the CD20 [73]. OCT2 and BOB1 immunohistochemistry performed in two cases showed expression in only one [73]. Unlike MCD, which is consistently IgM lambda-positive, GLPD plasmablasts often express monotypic kappa or lambda light chains, though five cases lacked identifiable immunoglobulin expression [72].

There are no established treatment guidelines for KSHV/HHV-8-positive GLPD, and management strategies are based largely on individual case reports and small case series. Zanelli et al. identified 19 published GLPD cases through the search date of their 2020 systematic review [72]. Seven patients were reported to have received chemotherapy alone; five achieved remission lasting 4–84 months, whereas two had refractory disease. Other reported approaches included observation, surgical excision, radiotherapy, and combined-modality treatment. Because treatment selection, response assessment, and follow-up were heterogeneous, these observations do not permit reliable comparison of treatment efficacy.

In the literature, some notable cases have been reported. A unique case reported by Martinez-Ciarpaglini et al. described a 79-year-old immunocompetent woman with localized axillary lymphadenopathy [76]. Unlike typical cases, GLPD in this patient exhibited a purely sinusoidal growth pattern without extrasinusoidal spread, mimicking anaplastic large cell lymphoma (ALCL) [76]. She responded well to Rituximab, Cyclophosphamide, Doxorubicin, Vincristine, and Prednisone (R-CHOP) therapy and remains disease-free after eight years, the longest recorded follow-up for GLPD [76].

In another case, a 73-year-old HIV-negative man presented with a left para-aortic mass detected by PET-CT. Immunohistochemical analysis revealed Notch1 and PD-L1 positivity. The PD-L1 overexpression by tumor cells suggests a potential therapeutic role for PD-1/PD-L1 immunotherapy, although further research is needed [77].

Reported clonality findings were heterogeneous, with most analysed lesions described as polyclonal and a smaller subset as oligoclonal. Next-generation sequencing performed in a limited number of cases did not identify established pathogenic driver mutations [72,73].

To summarize, KSHV/HHV-8-positive GLPD is an exceedingly rare HHV-8/EBV co-positive LPD that primarily affects germinal centers. While generally slow-growing, the disease may progress to high-grade lymphoma in some cases. Histopathological and immunophenotypic findings can differentiate KSHV/HHV-8-positive GLPD from similar disorders like MCD. Due to its rarity, no standardized treatment exists, and the comparative efficacy of chemotherapy, surgery, and radiotherapy cannot be determined from the available heterogeneous reports. Further studies, including investigations into immune checkpoint inhibitors, are necessary to optimize treatment approaches for KSHV/HHV-8-positive GLPD.

### 4.5. Emerging KSHV/HHV8–EBV Dual-Positive Large-Cell Lymphomas

In addition to PEL and KSHV/HHV8-positive GLPD, accumulating reports support the emergence of a third provisional group of KSHV/HHV8–EBV dual-positive large-cell lymphomas [11,15,57,58,59,60,61,62,76]. These are predominantly tissue-based lesions in which LANA-1 and EBER are demonstrated within the same large lesional-cell population, but the overall clinicopathological features do not fully satisfy the criteria for PEL, GLPD, or conventional KSHV/HHV8-positive diffuse large B-cell lymphoma. Although this group is not currently recognized as an independent WHO-HAEM5 or ICC entity, its recurring features justify collective consideration rather than treatment as unrelated case reports.

Wei Wang et al. reported two rare cases of large B-cell lymphoma (LBCL) co-infected with HHV-8 and EBV [59]. These cases exhibited both shared and unique features compared to PEL, KSHV/HHV-8-positive GLPD, and HHV-8-associated MCD. The first patient, a 61-year-old immunocompetent man with localized axillary lymphadenopathy, exhibited histological features reminiscent of Castleman disease, with tumor cells surrounding germinal centers and disrupting mantle zones. Immunophenotypically, the lymphoma was CD20+ and CD138−, with a high Ki-67 proliferation index and EBER positivity, distinguishing it from PEL, which lacks CD20 expression and occurs in immunosuppressed individuals, and KSHV/HHV-8-positive GLPD, where lymphoma cells remain confined to germinal centers [59]. The second patient, a 67-year-old woman, had widespread lymphadenopathy and rapid disease progression. Her lymphoma demonstrated a diffuse growth pattern with sinusoidal infiltration, immunoblastic/plasmablastic morphology, and high-grade features. Immunohistochemistry showed weak CD20 and CD45 expression, partial CD138 positivity, and co-expression of HHV-8 and EBER. Unlike typical solid PEL, this case lacked HIV positivity and exhibited unique characteristics, ultimately leading to the death of the patient within two weeks [59].

Ferry et al. documented two HHV-8-positive LPDs with distinctive pathological features [57]. The first case, a 61-year-old immunocompetent man with cervical and supraclavicular lymphadenopathy, had histological features resembling classical Hodgkin’s lymphoma but lacked its immunophenotypic markers [57]. His neoplastic cells were co-infected with HHV-8 and EBV, and he achieved complete remission after six cycles of R-CHOP chemotherapy [57]. The second case involved a 59-year-old HIV-positive Filipino man with an HHV8-positive but EBER-negative intravascular large B-cell lymphoma; it therefore represents a morphological comparator rather than a member of the provisional dual-positive group [57].

Additional reports describe rare cases of HHV-8+/EBV+ LBCLs. Crane et al. noted an HIV-positive patient with multiple subcutaneous nodules and HHV-8+/EBV+ intravascular lymphoma [61]. Similarly, cases of HHV-8+/EBV+ MCD have been documented, highlighting the variability of these disorders [59]. Papoudou-Bai et al. described an LBCL arising in MCD with unique features, including HIV seronegativity, EBV positivity, and absence of cytoplasmic immunoglobulin expression, a rare presentation since most HHV-8+ MCD-associated LBCLs are EBV-negative and occur in HIV-positive individuals [11]. Histopathology revealed large lymphoid cell clusters replacing follicular structures, consistent with MCD-associated lymphoma evolution [11].

A particularly notable case of HHV-8+/EBV+ MCD-associated plasmablastic microlymphoma involved a 45-year-old HIV-positive man whose cervical and inguinal lymph nodes exhibited MCD features with large plasmablasts replacing follicles [62]. Immunohistochemical analysis confirmed that large lymphoma cells were HHV-8, EBV, and MUM1 positive, with only focal λ light chain expression, but were negative for CD138 and immunoglobulin heavy chains [62]. Initially suspected as KSHV/HHV-8-positive GLPD, the case was reclassified as multifocal HHV-8+/EBV+ plasmablastic microlymphoma due to extensive disease, aggressive progression, and postmortem evidence of overt lymphoma [62]. Similar cases indicate that KSHV/HHV-8-positive GLPD may rarely progress to aggressive lymphoma in both HIV-positive and HIV-negative individuals [11,62].

Recent reports further illustrate the complexity of HHV-8+/EBV+ LPDs. One case of extracavitary nodal PEL evolved into pleural PEL, with focal intrasinusoidal tumor cell localization and Castleman-like histopathology [10]. Another case exhibited a spectrum of HHV-8-associated disorders—MCD, KSHV/HHV-8-positive GLPD, and PEL—coexisting within a single lymph node, highlighting the variability, overlap, and potential evolution among these entities [10]. Immunohistochemical, EBV latency, and molecular clonality studies confirmed these findings, yet both patients experienced unexpectedly indolent disease courses, surviving 8 and 12 months, respectively [10].

Carbone A. et al. reported an EBV-positive, HHV-8-associated lymphoma case in a 52-year-old male who presented with generalized lymphadenopathy [15]. Histologically, the lymphoma displayed an anaplastic large cell morphology with plasmablastic features, characterized by large pleomorphic cells with prominent nucleoli. Immunophenotypically, the tumor cells were positive for MUM1/IRF4, CD138, and CD30 but lacked expression of B-cell (CD20, PAX5) and T-cell (CD3) markers. In situ hybridization confirmed EBV positivity (EBER+), and HHV-8 LANA-1 was strongly expressed [15]. Molecular studies did not detect an immunoglobulin gene rearrangement; however, this finding alone does not establish a non-B-cell origin. The case underscores the expanding spectrum of HHV-8-associated lymphomas, suggesting a possible cooperative role of EBV and HHV-8 in lymphomagenesis, particularly in HIV-negative patients [15].

Collectively, these reports delineate an emerging group characterized by tissue-based large-cell proliferations with immunoblastic, plasmablastic, anaplastic, or Hodgkin-like morphology and same-cell KSHV/HHV8–EBV dual positivity. Nodal, sinusoidal, intravascular, and MCD-associated patterns have been described, while B-cell and plasma-cell marker expression is variable. Clinical behaviour is heterogeneous but may be aggressive. These recurring features support consideration of these lesions as a provisional third group of KSHV/HHV8–EBV dual-positive lymphomas, alongside PEL and GLPD. Nevertheless, the limited number of cases and absence of comparative molecular studies currently preclude their recognition as a distinct WHO-HAEM5 or ICC entity.

## 5. Conclusions

HHV-8, or KSHV, is a gamma-2 herpesvirus implicated in several lymphoproliferative disorders, lymphomas, and Kaposi sarcoma. Although KSHV/HHV-8 and EBV have conventionally been described as alternating between latent and lytic phases, their viral gene-expression programmes are not strictly binary. During abortive lytic replication, selected immediate-early or early lytic genes may be expressed without completion of the full replication cascade or production of infectious virions. These lytic gene products may contribute to oncogenesis through direct effects on infected cells and through paracrine conditioning of the tumour microenvironment. Both viruses evade immune detection through complex mechanisms, enabling persistent infection and an increased risk of malignancy, especially in immunocompromised hosts. HHV-8 is implicated in a range of lymphoproliferative disorders and lymphomas, including PEL and its solid variant extracavitary PEL, MCD, DLBCL, not otherwise specified, and the rare KSHV/HHV-8-positive GLPD. Notably, in both PEL and KSHV/HHV-8-positive GLPD, the neoplastic cells are frequently co-infected with EBV. In addition to PEL and GLPD, accumulating reports delineate a provisional third group of tissue-based KSHV/HHV8–EBV dual-positive large-cell lymphomas; however, current evidence remains insufficient for recognition as a distinct WHO-HAEM5 or ICC entity. The detection of KSHV/HHV8 and EBV within the same lesional cell population raises the possibility of cooperative effects in selected lymphoproliferative disorders. Overall, experimental models support a causal and cooperative role for KSHV/HHV8 and EBV in selected settings, whereas their precise contributions should be established separately for each human dual-positive lymphoproliferative disorder. Nevertheless, dual positivity should be regarded as a pathological and virological descriptor rather than as an independent WHO-HAEM5 or ICC disease category. The reported atypical cases demonstrate genuine clinicopathological overlap but do not yet establish that PEL, GLPD, KSHV/HHV8-associated MCD, and KSHV/HHV8-positive DLBCL represent a single biological continuum. Future studies should document the cellular localization of both viruses; the immune-dysregulation setting; immunoglobulin expression and clonality; and when progression is proposed the molecular relationship between sequential lesions. Such an integrated approach may clarify whether individual dual-positive proliferations represent variants of recognized entities, biologically related stages of disease, or currently unclassifiable lesions.

## Figures and Tables

**Table 1:** Comparative clinicopathological features of KSHV/HHV8-associated lymphoproliferative disorders and lymphomas, with emphasis on EBV status.

Disorder and Classification Status	Typical Site	HIV/Immunological Setting	EBV Status in Lesional Cells	Histological Architecture	Key Immunophenotype	Clinical Behaviour and Relationship to Aggressive Lymphoma
KSHV/HHV8-associated multicentric Castleman disease; WHO-HAEM5 tumour-like lesion with B-cell predominance	Multiple lymph-node regions; spleen may be involved	Commonly HIV-associated, but also occurs in HIV-negative, usually older, patients	Usually EBER-negative in the LANA-1-positive plasmablasts; rare dual-positive cases reported	Castleman-type follicles with LANA-1-positive plasmablasts, predominantly in mantle zones; interfollicular plasmacytosis and vascular proliferation	LANA-1+, MUM1/IRF4+, usually cytoplasmic IgMλ; EBER usually negative	Potentially severe cytokine-driven systemic disease; may coexist with or precede PEL or KSHV/HHV8-positive DLBCL
Primary effusion lymphoma (PEL); recognized aggressive lymphoma	Pleural, peritoneal, or pericardial cavities, usually without a solid mass	Strong association with HIV; also occurs in other immunodeficiency settings and in older HIV-negative patients	Frequently EBER-positive, particularly in HIV-associated PEL, but EBV is not required for diagnosis	Discohesive large plasmablastic, immunoblastic, or anaplastic cells in an effusion	LANA-1+; MUM1/IRF4+, CD38/CD138 and CD30 variably positive; conventional B-cell markers usually absent or weak; EBER variable	Aggressive lymphoma with generally poor prognosis; “progression to lymphoma” is not applicable
Extracavitary PEL (EC-PEL); solid-tissue presentation of PEL rather than a separate entity	Lymph nodes, gastrointestinal tract, skin, and other extranodal sites	Similar to PEL	Frequently EBER-positive but EBV is not required for diagnosis	Solid or diffuse tissue infiltrate of plasmablastic or immunoblastic cells	Similar to PEL; LANA-1+, plasma-cell-associated phenotype; conventional B-cell markers usually absent or weak	Aggressive lymphoma; may coexist with, precede, or follow cavity-based PEL
KSHV/HHV8-positive germinotropic lymphoproliferative disorder (GLPD); recognized lymphoproliferative disorder	Usually localized lymph nodes, particularly in the head and neck	Usually HIV-negative; rare HIV-positive cases	Characteristically EBER-positive; rare EBV-negative cases have been described	Plasmablasts preferentially infiltrate or replace germinal centres, often with relatively preserved nodal architecture	LANA-1+ and usually EBER+; cytoplasmic immunoglobulin commonly monotypic; conventional B-cell markers often absent or weak; MUM1/IRF4, CD38, and CD30 variable; CD138 usually negative	Indolent in most reported cases, although persistent, disseminated, and progressive cases occur; rare progression to large-cell lymphoma
KSHV/HHV8-positive diffuse large B-cell lymphoma; recognized aggressive lymphoma	Usually lymph nodes and spleen, often arising in association with KSHV/HHV8-associated MCD	Commonly associated with HIV or another immunodeficiency setting	Typically EBER-negative; EBV-positive cases require careful exclusion of EC-PEL, GLPD, and the emerging KSHV/HHV8–EBV dual-positive large-cell lymphoma group	Diffuse sheets of plasmablasts, sometimes arising in a background of MCD	LANA-1+, MUM1/IRF4+, commonly cytoplasmic IgMλ; conventional B-cell markers frequently absent or weak; EBER usually negative	Aggressive lymphoma with poor prognosis; “progression to lymphoma” is not applicable
Emerging KSHV/HHV8–EBV dual-positive large-cell lymphoma; provisional group, not a formal WHO-HAEM5 or ICC entity	Variable; lymph nodes, skin or subcutaneous tissue, extranodal sites, sinusoids, or vessels	Variable; reported in both HIV-positive and HIV-negative individuals	Positive by definition, with LANA-1 and EBER demonstrated within the same morphologically defined large lesional-cell population	Diffuse, perifollicular or mantle-zone, sinusoidal, or intravascular patterns; an MCD-like background may be present	LANA-1+ and EBER+ in the same large lesional cells; MUM1/IRF4 commonly expressed; CD20, PAX5, CD79a, CD30, CD38, CD138, and immunoglobulin variably expressed	Clinical behaviour is heterogeneous but may be aggressive; reported outcomes range from rapid progression to complete remission or, less commonly, an indolent course. The limited number of cases precludes reliable estimation of prognosis or treatment efficacy

Abbreviations: DLBCL, diffuse large B-cell lymphoma; EBV, Epstein–Barr virus; EBER, EBV-encoded RNA; EC-PEL, extracavitary primary effusion lymphoma; GLPD, germinotropic lymphoproliferative disorder; KSHV/HHV8, Kaposi sarcoma-associated herpesvirus/human herpesvirus 8; LANA-1, latency-associated nuclear antigen 1; MCD, multicentric Castleman disease; PEL, primary effusion lymphoma.

## Data Availability

No new data were created or analyzed in this study. Data sharing is not applicable to this article.
